# Remarkable Response of Hairy Cell Leukemia Variant to Single-Agent Cladribine

**DOI:** 10.7759/cureus.24976

**Published:** 2022-05-13

**Authors:** Steve B Otieno, Osarenren Ogbeide

**Affiliations:** 1 Hematology/Oncology, University of Tennessee Health Science Center, Memphis, USA; 2 Hematology/Oncology, Veteran's Affairs Hospital, Memphis, USA

**Keywords:** hairy cell leukemia variant, brafv600e, splenomegaly, purine analog, cytopenias

## Abstract

Classical hairy cell leukemia (cHCL) and related mature lymphoid B-cell neoplasms including hairy cell leukemia variant (HCLv) and splenic diffuse red pulp lymphoma (SDRPL) are a rare subset of lymphoid neoplasms. cHCL accounts for around 2% of all leukemias and is characterized by a peripheral smear with large lymphoid cells with cytoplasmic projections giving the cells a hairy appearance, splenomegaly, and cytopenias. Majority of cHCL cases harbor a BRAFV600E mutation. cHCL usually responds well to single-agent purine analogs. HCLv is even rarer and constitutes around 0.4% of lymphoid malignancies. Unlike cHCL, HCLv is less responsive to standard single-agent purine analogs and typically does not harbor the BRAFV600E mutation. The “hairy cells,” splenomegaly, and cytopenias are common in both. We report a case of a patient with HCLv who was treated with a single purine analog and achieved a near-complete response.

## Introduction

Hairy cell leukemia variant (HCLv) is a rare B-cell lymphoproliferative disorder with malignant cells that are morphologically similar to classical hairy cell leukemia (cHCL) but is a clinically and biologically distinct entity [1]. Morphologically, both disorders are characterized by mature B-cells with abundant cytoplasm and hairy projections in the peripheral blood, bone marrow, and spleen. Both disorders are also typically associated with splenomegaly due to the accumulation of malignant cells in the spleen. However, molecular evaluation typically reveals a BRAFV600E mutation in the cHCL but not in the HCLv. In terms of clinical presentation, HCLv has a more aggressive clinical course relative to cHCL and is usually either primary refractory to standard therapy or poorly responsive. While cHCL has response rates of greater than 90%, with majority being complete responses when treated with the standard single-agent purine analogs cladribine and pentostatin, HCLv has much lower response rates, in the 40-50% range, with the majority of responses being partial reponses [2,3]. Better responses in small studies have been observed with the addition of rituximab to the purine analogs [4]. One small study of 10 patients reported a 90% response rate with cladribine and rituximab [4].

We report a case of a patient with HCLv who was treated with single-agent cladribine and achieved a near-complete response.

## Case presentation

The patient is a 68-year-old male who was advised to go to the Veterans Affairs Emergency Department by his primary care doctor after he was noted to have a white blood cell (WBC) count of 28 x 10^3^ per microliter (µL). His past medical history was notable for a bronchogenic cyst status post-resection six years prior to presentation, hypertension, hyperlipidemia, and a remote history of rheumatic fever and malaria greater than four decades prior to presentation. He reported that over the month prior to presentation, he had been having a productive cough and a decreased appetite, and had lost about 10 pounds. He denied fever, chills, chest pain, shortness of breath, abdominal pain, or changes in bowel or urinary habits. For medication, he was taking amlodipine and atorvastatin. He did not have a family history of malignancy or blood disorders. He was a smoker with a 42 pack-year smoking history. He had a history of heavy alcohol use but had been abstinent for a few years and denied any illicit drug use.

**Figure 1:**
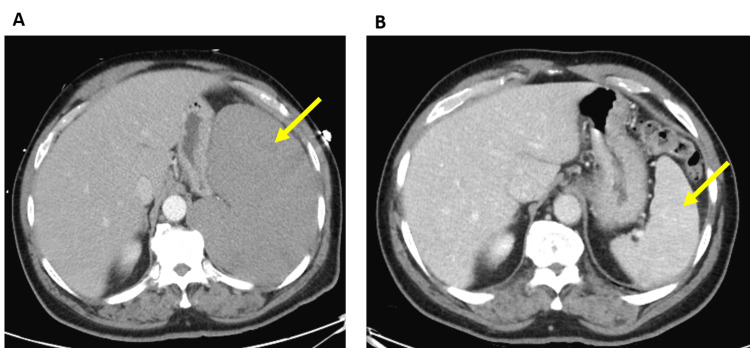
Resolution of splenomegaly with treatment. Spleen marked by yellow arrows. (A) Before treatment. (B) Five months after treatment.

On examination, he had a temperature of 98.6˚F, a heart rate of 78 beats per minute, a respiratory rate of 14 breaths per minute, and a blood pressure of 150 mmHg/92 mmHg. On examination, he was in no acute distress and was an obese male with a body mass index (BMI) of 33 kg/m^2^. Physical examination was notable for wheezes in lung fields bilaterally and splenomegaly. No cervical, axillary or inguinal adenopathy was noted. Laboratories were notable for a WBC count of 28.65 x 10^3^ per µL, an absolute neutrophil count (ANC) of 4.53 x 10^3^ per µL, a hemoglobin level (Hgb) of 12.9 grams per deciliter (g/dL), and a platelet count of 95 x 10^3^ per µL. Electrolytes, liver function tests, and renal function were within normal limits. Computed tomography (CT) of the chest with contrast showed a small left-sided effusion with a left lower lobe atelectasis versus consolidation. The visualized portions of the abdomen showed splenomegaly and enlarged gastro-hepatic ligament nodes. A follow-up CT of the abdomen was notable for marked splenomegaly with a spleen span of 21 cm (Figure 1). He was admitted and started on treatment for community-acquired pneumonia and further evaluation. Due to the weight loss, leukocytosis, splenomegaly, and adenopathy, a peripheral smear was ordered, and the hematology/oncology service was consulted for concern of a hematologic malignancy.

**Figure 2:**
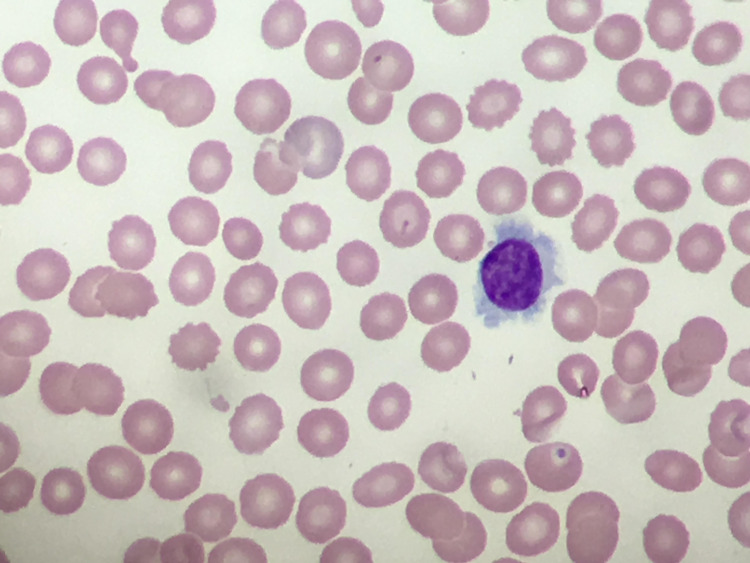
Hairy cell leukemia variant cell on peripheral smear characterized by atypical lymphocytes with cytoplasmic projections

A review of the peripheral smear was notable for lymphocytosis with atypical lymphocytes with thin cytoplasmic projections (Figure 2). In the context of splenomegaly, the differential diagnoses were hairy cell leukemia versus splenic marginal zone lymphoma. Clinically, the patient continued to improve and was discharged a day after admission with a plan for an outpatient bone marrow biopsy. A bone marrow biopsy was performed six days after discharge.

Complete blood count (CBC) done on the day of the bone marrow biopsy showed a WBC count of 39.26 x 10^3^ per µL, ANC of 3.36 x 10^3^ per µL, Hgb of 12.0 g/dL, and a platelet count of 113 x 10^3^ per µL. The aspirate showed normoblastic myeloid and erythroid maturation without significant dyspoeitic features. There was no increase in blasts. Atypical lymphocytes morphologically similar to those seen on the peripheral smear were seen. The core biopsy and clot sections showed cellularity of 20-40%. A reticulin stain showed mild, focal increase in reticulin fibers (grade 1). cHCL is typically positive for CD25 and CD103 and HCLv is negative for CD25 but may be positive for CD103. Immunophenotyping showed B-lymphocytes that were positive for CD19, CD20, had a moderate expression of CD200, and had a bright CD11c and CD103. They were negative for expression of surface kappa and lambda light chain, CD5, CD10, CD38, CD23, and CD25. Fluorescence in situ hybridization was negative for BCR/ABL translocation. Cytogenetics detected two separate complex karyotypes involving multiple deletions, monosomy 5, and an apparent derivative of chromosome 16. Molecular studies were negative for BRAFV600E mutation. The most consistent diagnosis was HCLv.

On the first clinic visit two weeks after the biopsy, his CBC showed a WBC count of 48 x 10^3^ per µL, ANC of 5.32 x 10^3^ per µL, Hgb of 11.5 g/dL, and a platelet count of 103 x 10^3^ per µL. He was doing well with an Eastern Cooperative Oncology Group (ECOG) functional status of 1. Per the National Comprehensive Cancer Network (NCCN) guidelines, he did not have indication for treatment at that time. We discussed the options of management including observation, treatment with cladribine or pentostatin, or treatment with cladribine or pentostatin with addition of rituximab. The patient and family decided on observation with close follow-up with a scan in the interim.

On the three-week follow-up visit, he complained of left-sided abdominal pain and that he could no longer lie flat and had to sit on a recliner to sleep. The interim MRI showed worsening splenomegaly with a spleen size now increased to 23 cm from 21 cm as well as splenic infarcts and worsening abdominal adenopathy. A CBC showed a WBC count of 54 x 10^3^ per µL, ANC of 4.59 x 10^3^ per µL, Hgb of 11.7 g/dL, and a platelet count of 81 x 10^3^ per µL. We recommended starting treatment given these findings. The patient was decided on cladribine with a plan for weekly rituximab four weeks after completion of cladribine for a total of eight weeks. CBC done before treatment showed a WBC count of 66 x 10^3^ per µL, ANC of 4.1 x 10^3^ per µL, Hgb of 10.4 g/dL, and a platelet count of 95 x 10^3^ per µL (Table 1).

**Table 1:** Laboratory Values

	Units	Normal Range	Before Treatment	12 Days After Treatment	4.5 Months After Treatment
White blood cell count	10^3^/µL	4-10	66.13	1.98	4.79
Absolute neutrophil count	10^3^/µL	1.5-7.4	4.1	0.66	3.24
Hemoglobin	g/dL	13.5-17	10.4	9.0	14.1
Platelets	10^3^/µL	150-420	95	63	157

He was started on treatment with single-agent cladribine. He was treated with 0.12 milligrams per kilogram per day on days 1-5. His treatment was complicated by febrile neutropenia requiring admission on day 11 with an ANC nadir of 0.66 x 10^3^ per µL, multifocal pneumonia, and influenza B infection. His other blood counts were WBC count of 1.98 x 10^3^ per µL, Hgb of 9.0 g/dL, and a platelet count of 63 x 10^3^ per µL (Table 1). He was treated with broad-spectrum antibiotics and oseltamivir. His ANC improved without requiring granulocyte colony-stimulating factor (G-CSF). Given the complication of infection and general decline in the patient’s functional status, the patient decided against getting the rituximab.

On the first clinic visit after discharge, which was day 22 since starting chemotherapy, he was now able to lie flat and his breathing had improved considerably. On examination, his previous massive splenomegaly had resolved. CBC showed a WBC count of 2.54 x 10^3^ per µL, ANC of 1.11 x 10^3^ per µL, Hgb of 10.8 g/dL, and a platelet count of 157 x 10^3^ per µL. He was seen monthly in the interim with blood counts improving to within normal range. Response was evaluated about 4.5 months after completion of therapy. No splenomegaly or lymphadenopathy was noted on examination, and CBC showed a WBC count of 4.79 x 10^3^ per µL, ANC of 3.74 x 10^3^ per mL, Hgb of 14.1 g/dL, and a platelet count of 157 x 10^3^ per µL, all normal (Table 1). Review of peripheral smear showed no residual hairy leukemia cells by morphology or by flow cytometry. A bone marrow biopsy showed a hypocellular marrow for age with 10-15% cellularity with trilineage maturation and no morphologic or immunophenotypic evidence or residual hairy cell leukemia. Grade 3 reticulin fibrosis was noted. A CT of the chest, abdomen, and pelvis showed complete resolution of splenomegaly with old infracts noted and almost complete resolution of upper abdominal adenopathy with only a single node, which was markedly reduced in size remaining. He continues to do well and has normal blood counts (Figure 1).

## Discussion

Treatment of HCLv remains a challenge. Response to single-agent purine analogs cladribine and pentostatin, which are very effective in treating cHCL, is poor. Current expert opinions suggest the use of purine analogs plus rituximab [5]. The patient in this report presented with leukocytosis, mild thrombocytopenia, and splenomegaly, and was found to have HCLv. The initial plan was to treat with cladribine and rituximab in a sequential manner. After receiving cladribine, he developed multifocal pneumonia and influenza and had a general decline in functional status. The patient and family decided against further therapy. His pancytopenia and splenomegaly resolved, and a follow-up bone marrow 4.5 months after treatment showed no residual HCLv cells.

The patient in this case represents the small proportion of HCLv patients who achieve complete or near-complete responses with single-agent purine analogs. The predictive markers for this subset of patients remain unknown. How long such responses last and overall survival in this population is still unclear. Further studies will be needed to validate this.

The toxicities of doublet therapy with rituximab in a large study is still unclear. There are currently two clinical trials (NCT00923013 and NCT02157181) looking into the effectiveness and toxicities of cladribine plus rituximab in the front line and relapsed/refractory setting. Determination of patients who may have good outcomes with single-agent therapy may spare such patients of toxicities of the doublet regimen as well allow for a shorter treatment duration. While treatment with cladribine takes around only one week to complete, addition of rituximab can add on several weeks to months.

## Conclusions

HCLv typically has a poorer response to single-agent purine analogs relative to the cHCL. However, there is a population of HCLv patients, like the patient in this case, who have a good response to single-agent purine analogs. Markers for this population of patients may potentially spare them toxicities of double therapy. Trials are ongoing to study the effectiveness and toxicities of cladribine plus rituximab in the front line and relapsed/refractory setting.
